# Synthesis and crystal structure of di­aqua­bis­(hexa­methyl­enetramine-κ*N*)bis­(thio­cyanato-κ*N*)cobalt(II)–hexa­methyl­ene­tetra­mine–aceto­nitrile (1/2/2)

**DOI:** 10.1107/S2056989021010033

**Published:** 2021-10-08

**Authors:** Christoph Krebs, Magdalena Ceglarska, Christian Näther

**Affiliations:** a Institut für Anorganische Chemie, Christian-Albrechts-Universität zu Kiel, Max-Eyth-Str. 2, D-24118 Kiel, Germany; b Institute of Physics, Jagiellonian University, Lojasiewicza 11, 30-348 Kraków, Poland

**Keywords:** crystal structure, cobalt thio­cyanate, hexa­methyl­ene­tetra­mine, discrete complex, hydrogen bonding

## Abstract

The crystal structure of the title solvated coordination compound, [Co(NCS)_2_(C_6_H_12_N_4_)_2_(H_2_O)_2_]·2C_6_H_12_N_4_·2C_2_H_3_N, consists of discrete complexes in which the Co^2+^ cations (site symmetry  ) are sixfold coordinated by two N-bonded thio­cyanate anions, two water mol­ecules and two hexa­methyl­ene­tetra­mine (HMT) mol­ecules to generate distorted *trans*-CoN_4_O_2_ octa­hedra. The discrete complexes are each connected by two HMT solvate mol­ecules into chains *via* strong O—H⋯N hydrogen bonds. These chains are further linked by additional O—H⋯N and C—H⋯N and C—H⋯S hydrogen bonds into a three-dimensional network. Within this network, channels are formed that propagate along the *c*-axis direction and in which additional aceto­nitrile solvent mol­ecules are embedded, which are hydrogen bonded to the network. The CN stretching vibration of the thio­cyanate ion occurs at 2062 cm^−1^, which is in agreement with the presence of N-bonded anionic ligands. XRPD investigations prove the formation of the title compound as the major phase accompanied by a small amount of a second unknown phase.

## Abstract 2

The crystal structure of the title compound consists of discrete neutral complexes in which the cobalt cations are octa­hedrally coordinated by two N-bonded thio­cyanate anions, two hexa­methyl­ene­tetra­amine ligands and two water mol­ecules with additional aceto­nitrile and hexa­methyl­ene­tetra­mine solvate mol­ecules, which are hydrogen bonded to the complexes.

## Chemical context

For several years, we have been inter­ested in the synthesis of coordination compounds based on cobalt thio­cyanate and additional co-ligands that in most cases consist of N-donor ligands. As is the case for, *e.g.* cyanides and azides, even this anionic ligand is able to mediate reasonable magnetic exchange (Mekuimemba *et al.*, 2018 ▸; Mousavi *et al.*, 2020 ▸; Palion-Gazda *et al.*, 2015 ▸). Therefore, we have focused especially on compounds in which the metal cations are linked by anionic ligands into coordination polymers. Most of the compounds with monocoordinating co-ligands consist of linear chains and show anti­ferromagnetic or ferromagnetic ordering or are single-chain magnets (Shi *et al.*, 2006 ▸; Jin *et al.*, 2007 ▸; Prananto *et al.*, 2017 ▸; Mautner *et al.*, 2018 ▸; Rams *et al.*, 2020 ▸; Ceglarska *et al.*, 2021 ▸; Werner *et al.*, 2014 ▸, 2015 ▸), whereas in compounds with non-linear chains the magnetic exchange is completely suppressed (Böhme *et al.*, 2020 ▸). In some cases, layered compounds are obtained, that are exclusively ferromagnets (Suckert *et al.*, 2016 ▸; Wellm *et al.*, 2020 ▸). All these compounds have in common that only monocoordinating co-ligands are used, which means that the thio­cyanate substructures are not additionally connected into structures of higher dimensionality. We have therefore tried to link the Co(NCS)_2_ chains or layers by bridging co-ligands.

In this context, we became inter­ested in urotropine, C_6_H_12_N_4_ (also called hexa­methyl­ene­tetra­mine or 1,3,5,7-tetra­aza­adamantane), as a co-ligand. On one hand, this ligand is magnetically silent and on the other hand it is able to form tetra­hedral networks and some examples have been reported in the literature (Czubacka *et al.*, 2012 ▸; Li *et al.*, 2012 ▸). It is noted that some compounds with this ligand and Co(NCS)_2_ have already been reported in the literature. In all cases, discrete complexes are formed in which the cobalt cations are octa­hedrally coordinated by two thio­cyanate anions and some water, methanol or urotropine ligands (see *Database survey*). Compounds with urotropine in which the cobalt cations are linked by bridging thio­cyanate anions have not been reported. 
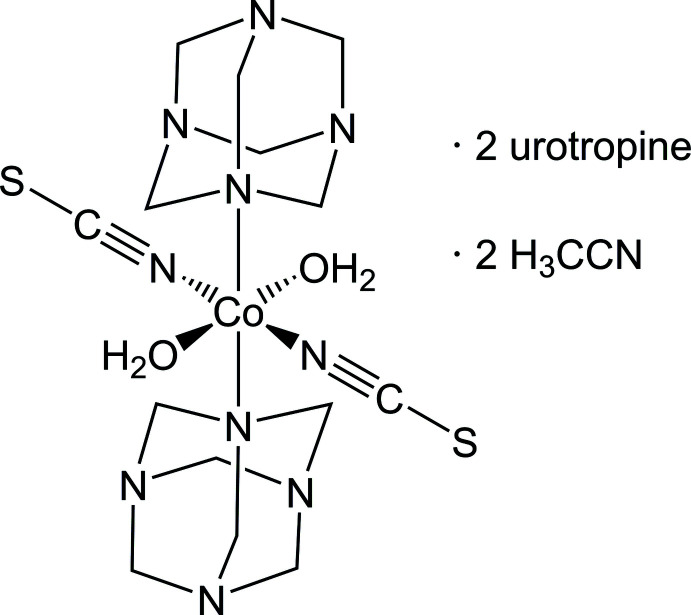


In the course of this project, we reacted Co(NCS)_2_ with urotropine in aceto­nitrile, resulting in the formation of a light-yellow-colored crystalline phase, for which IR spectroscopic investigations revealed the CN stretching vibration to be 2062 cm^−1^. This indicates the presence of only N-bonded thio­cyanate anions (see Fig. S1 in the supporting information). To identify this compound, a single-crystal structure analysis was performed, which proves that a discrete complex has formed. Comparison of the X-ray powder pattern of this crystalline phase with that calculated from single-crystal data reveals that the title compound has formed as the major phase, but that there are still some reflections indicating the formation of an additional and unknown crystalline phase (Fig. S2).

## Structural commentary

**Figure 1:**
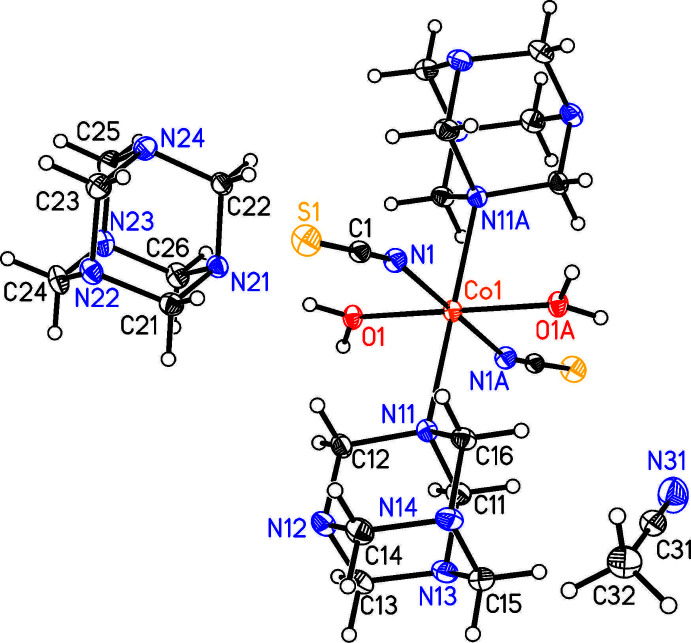
Crystal structure of the title compound with atom labeling and displacement ellipsoids drawn at the 50% probability level. Symmetry operation for the generation of equivalent atoms: (A) −*x* + 1, −*y* + 1, −*z* + 1.

In the crystal structure of the title compound, [Co(NCS)_2_(H_2_O)_2_(C_6_H_12_N_4_)_2_]·(C_6_H_12_N_4_)_2_(C_2_H_3_N)_2_, the cobalt cations are each octa­hedrally coordinated by two N-bonded thio­cyanate anions, two urotropine mol­ecules and two water mol­ecules to form discrete complexes that are located on centers of inversion (Fig. 1 ▸). The Co1—O1 and the thio­cyanate Co1—N1 bond lengths are similar, whereas the Co1—N11 bond length to the neutral co-ligand is significantly longer (Table 1 ▸). The *cis*-angles around the Co centers deviate from ideal values, showing that the octa­hedra are slightly distorted [range of *cis* bond angles = 87.51 (4)–92.49 (4)°]. This is also apparent from the value of the octa­hedral angle variance of 2.540°^2^ and the mean octa­hedral quadratic elong­ation of 1.006 calculated by the method of Robinson *et al.* (1971 ▸).

## Supra­molecular features

**Figure 2:**
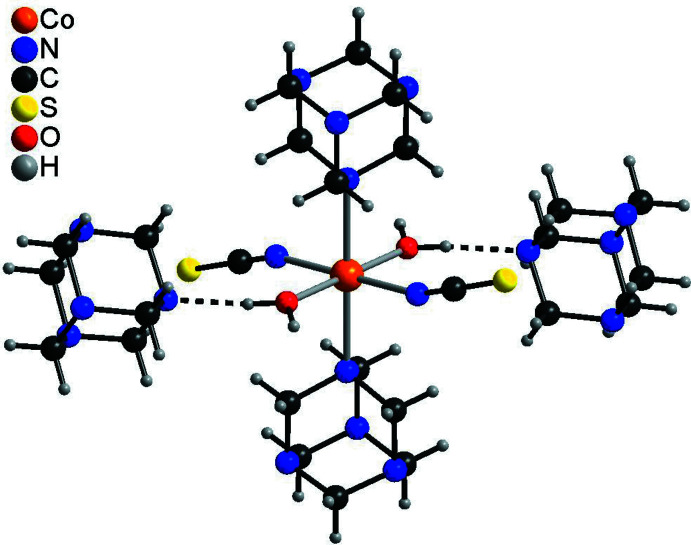
View of a discrete complex that is connected to two hexa­methyl­enetramine solvent mol­ecules *via* O—H⋯N hydrogen bonds (dashed lines).

**Figure 3:**
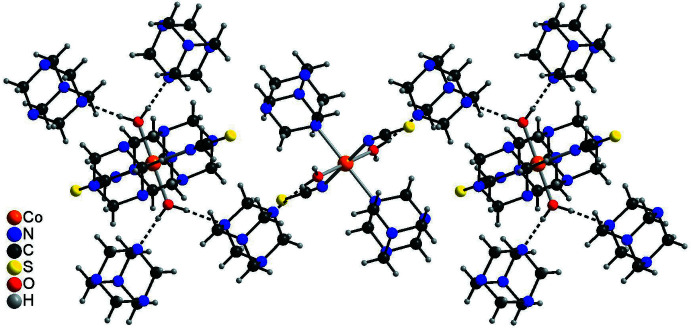
Part of the crystal structure of the title compound showing the connection of discrete complexes by hexa­methyl­enetramine solvate mol­ecules *via* O—H⋯N hydrogen bonds (dashed lines).

**Figure 4:**
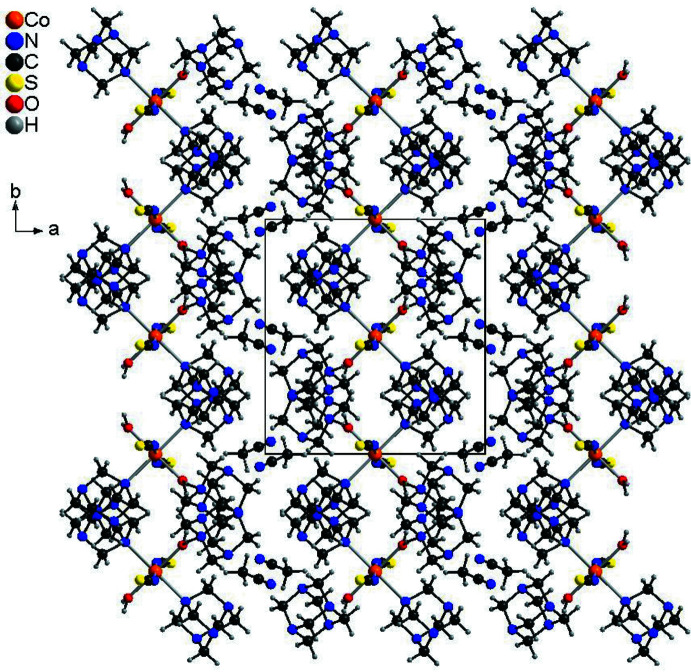
Crystal structure of the title compound viewed along the *c*-axis.

The crystal structure of the title compound is dominated by a variety of inter­molecular O—H⋯N, C—H⋯N and C—H⋯S hydrogen bonds (Table 2 ▸). Each complex mol­ecule is connected to two adjacent non-coordinating urotropine solvate mol­ecules *via* O—H⋯N hydrogen bonds from one of the water H atoms. The O—H⋯N angle is close to linear and the N⋯H distance amounts to 1.85 (2) Å, which indicates a very strong inter­action (Fig. 2 ▸). The complex mol­ecules are linked by the urotropine solvate mol­ecules into chains (Fig. 3 ▸). The chains are further connected by an O—H⋯N hydrogen bond arising from the second water hydrogen atom into layers, and these layers are further linked into a three-dimensional network by a number of weak C—H⋯N and C—H⋯S hydrogen bonds. In this way, channels are formed along the crystallographic *c*-axis direction in which additional aceto­nitrile mol­ecules are located (Fig. 4 ▸). These mol­ecules are linked to the main network *via* C—H⋯N inter­actions (Table 2 ▸).

## Database survey

Some crystal structures have already been deposited in the Cambridge Structure Database (CSD version 5.42, last update November 2020; Groom *et al.*, 2016 ▸) that contain cobalt cations, thio­cyanate anions and urotropine mol­ecules. These include [Co(NCS)_2_(C_6_H_12_N_4_)(CH_3_OH)_2_(H_2_O)] (refcode: POFGAT; Shang *et al.*, 2008 ▸), which consists of neutral complexes in which the cobalt cations are octa­hedrally coord­inated by the N atoms of two thio­cyanate anions, two methanol, one water and one urotropine ligand to generate a *mer*-CoN_3_O_3_ coordination polyhedron. [Co(NCS)_2_(H_2_O)_4_]·2C_6_H_12_N_4_ (XILXOG; Li *et al.*, 2007 ▸) is a discrete complex with a cobalt cation coordinated octa­hedrally by two thio­cyanate anions and four water ligands (as a *trans*-CoN_2_O_4_ octa­hedron) with two additional urotropine solvent mol­ecules. The structure of [Co(NCS)_2_(C_6_H_12_N_4_)_2_(H_2_O)_2_][Co(NCS)_2_(H_2_O)_4_]·2H_2_O has been determined several times (MOTNIS; Liu *et al.*, 2002 ▸; MOTNIS01; Zhang *et al.*, 1999 ▸; MOTNIS02; Chakraborty *et al.*, 2006 ▸; MOTNIS03; Lu *et al.*, 2010 ▸) and contains two discrete octa­hedral cobalt complexes: one metal ion is coord­inated by two thio­cyanate anions, two water mol­ecules and two urotropine mol­ecules (*trans*-CoN_4_O_2_ octa­hedron) and the other by two thio­cyanate anions and four water mol­ecules (*trans*-CoO_4_N_2_ octa­hedron); two water mol­ecules of crystallization complete the structure.

## Synthesis and crystallization

**Synthesis**

Co(NCS)_2_ and urotropine were purchased from Merck. All chemicals were used without further purification.

Light-yellow-colored single crystals suitable for single crystal X-ray analysis were obtained after heating 0.15 mmol Co(NCS)_2_ (26.3 mg) and 0.30 mmol urotropine (42.1 mg) in 0.5 ml MeCN up to 353 K and then storing the mixture at 333 K overnight.

Since it was not possible to obtain a crystalline powder of the title component from solution, a sample was taken from the single crystal batch, crushed and measured.

IR: ν = 2967 (*w*), 2958 (*sh*), 2930 (*sh*), 2920 (*w*), 2881 (*w*), 2309 (*vw*), 2281 (*w*), 2252 (*vw*), 2234 (*vw*), 2185 (*vw*), 2168 (*vw*), 2062 (*s*), 1952 (*vw*), 1684–1560 (*vw*), 1461 (*m*), 1417 (*sh*), 1378 (*w*), 1372 (*w*), 1363 (*w*), 1325 (*vw*), 1231 (*s*), 1049 (*w*), 994 (*vs*), 935 (*w*), 917 (*m*), 825 (*m*), 800 (*m*), 782 (*m*), 770 (*m*), 731 (*sh*), 690 (*s*), 662 (*s*), 506 (*m*) cm^−1^.

**Experimental details**

The data collection for the single-crystal structure analysis was performed using an XtaLAB Synergy, Dualflex, HyPix diffractometer from Rigaku with Cu *K*α radiation.

The PXRD measurement was performed with Cu *K*α_1_ radiation (λ = 1.540598 Å) using a Stoe transmission powder diffraction system (STADI P) equipped with a MYTHEN 1K detector and a Johansson-type Ge(111) monochromator.

The IR spectrum was measured using an ATI Mattson Genesis Series FTIR spectrometer, control software: WINFIRST, from ATI Mattson.

## Refinement

Crystal data, data collection and structure refinement details are summarized in Table 3 ▸. All non-hydrogen atoms were refined anisotropically. Water O atoms were freely refined. The C-bound H atoms were located in a difference map but positioned with idealized geometry (C—H = 0.96–0.97 Å, methyl H atoms allowed to rotate but not to tip) and were refined isotropically with *U*_iso_(H) = 1.2*U*_eq_(C) (1.5 for methyl H atoms) using a riding model.

## Supplementary Material

Crystal structure: contains datablock(s) I. DOI: 10.1107/S2056989021010033/hb7982sup1.cif

Structure factors: contains datablock(s) I. DOI: 10.1107/S2056989021010033/hb7982Isup2.hkl

Fig. S1. Experimental (top) and calculated X-ray powder pattern (bottom) of the title compound. The title compound is contaminated with at least one additional unknown phase. For the calculation of the powder pattern the data obtained from a a single crystal measured at 24 C was used. DOI: 10.1107/S2056989021010033/hb7982sup3.png

Fig. S2. IR spectra of the title compound. The value of the CN stretching vibration of the thiocyanat anions is given. DOI: 10.1107/S2056989021010033/hb7982sup4.png

CCDC reference: 2112185

Additional supporting information:  crystallographic information; 3D view; checkCIF report

## Figures and Tables

**Table 1:** Selected geometric parameters (Å, °)

Co1—N1	2.0744 (10)	Co1—N11	2.3112 (9)
Co1—O1	2.0661 (8)		
			
C1—N1—Co1	161.53 (9)		

**Table 2:** Hydrogen-bond geometry (Å, °)

*D*—H⋯*A*	*D*—H	H⋯*A*	*D*⋯*A*	*D*—H⋯*A*
O1—H1*A*⋯N21	0.88 (2)	1.85 (2)	2.7298 (13)	171.8 (18)
O1—H1*B*⋯N22^i^	0.88 (2)	2.01 (2)	2.8759 (13)	167.7 (19)
C12—H12*B*⋯O1	0.97	2.60	3.0752 (14)	111
C13—H13*A*⋯S1^i^	0.97	2.95	3.8089 (12)	148
C13—H13*B*⋯N24^ii^	0.97	2.66	3.5045 (16)	146
C16—H16*A*⋯O1^iii^	0.97	2.52	3.0571 (14)	115
C16—H16*B*⋯N1	0.97	2.70	3.2713 (15)	118
C21—H21*A*⋯S1	0.97	3.00	3.9471 (12)	165
C23—H23*B*⋯S1^iv^	0.97	2.89	3.6683 (12)	138
C26—H26*A*⋯N31^iii^	0.97	2.56	3.4794 (17)	158
C32—H32*A*⋯S1^v^	0.96	3.02	3.9560 (15)	166
C32—H32*B*⋯N23^vi^	0.96	2.58	3.4685 (16)	154
C32—H32*C*⋯N14^v^	0.96	2.61	3.4750 (17)	149

Symmetry codes: (i) x, -y+{\script{3\over 2}}, z-{\script{1\over 2}}; (ii) -x+1, y+{\script{1\over 2}}, -z+{\script{3\over 2}}; (iii) -x+1, -y+1, -z+1; (iv) -x+1, -y+1, -z+2; (v) -x, -y+1, -z+1; (vi) x-1, -y+{\script{3\over 2}}, z-{\script{1\over 2}}.

**Table 3:** Experimental details

Crystal data	
Chemical formula	[Co(NCS)_2_(C_6_H_12_N_4_)_2_(H_2_O)_2_]·2C_6_H_12_N_4_·2C_2_H_3_N
*M*_r_	854.01
Crystal system, space group	Monoclinic, *P*2_1_/*c*
Temperature (K)	100
*a*, *b*, *c* (Å)	13.0008 (2), 12.5903 (2), 12.9988 (2)
β (°)	114.899 (2)
*V* (Å^3^)	1929.93 (6)
*Z*	2
Radiation type	Cu *K*α
μ (mm^−1^)	4.99
Crystal size (mm)	0.20 × 0.04 × 0.03

Data collection	
Diffractometer	XtaLAB Synergy, Dualflex, HyPix
Absorption correction	Multi-scan (*CrysAlis PRO*; Rigaku OD, 2021 ▸)
*T*_min_, *T*_max_	0.779, 1.000
No. of measured, independent and observed [*I* > 2σ(*I*)] reflections	25388, 4088, 3972
*R*_int_	0.021
(sin θ/λ)_max_ (Å^−1^)	0.635

Refinement	
*R*[*F* ^2^ > 2σ(*F* ^2^)], *wR*(*F* ^2^), *S*	0.025, 0.071, 1.09
No. of reflections	4088
No. of parameters	259
H-atom treatment	H atoms treated by a mixture of independent and constrained refinement
Δρ_max_, Δρ_min_ (e Å^−3^)	0.20, −0.39

Computer programs: *CrysAlis PRO* (Rigaku OD, 2021 ▸), *SHELXT2014/5* (Sheldrick, 2015*a* ▸), *SHELXL2016/6* (Sheldrick, 2015*b* ▸), *DIAMOND* (Brandenburg & Putz, 1999 ▸) and *publCIF* (Westrip, 2010 ▸).

## supplementary crystallographic information

### Crystal data

**Table:**

[Co(NCS)_2_(C_6_H_12_N_4_)_2_(H_2_O)_2_]·2C_6_H_12_N_4_·2C_2_H_3_N	*F*(000) = 906
*M**_r_* = 854.01	*D*_x_ = 1.470 Mg m^−^^3^
Monoclinic, *P*2_1_/*c*	Cu *K*α radiation, λ = 1.54178 Å
*a* = 13.0008 (2) Å	Cell parameters from 20611 reflections
*b* = 12.5903 (2) Å	θ = 3.7–77.9°
*c* = 12.9988 (2) Å	µ = 4.99 mm^−^^1^
β = 114.899 (2)°	*T* = 100 K
*V* = 1929.93 (6) Å^3^	Needle, light yellow
*Z* = 2	0.20 × 0.04 × 0.03 mm

### Data collection

**Table:**

XtaLAB Synergy, Dualflex, HyPix diffractometer	4088 independent reflections
Radiation source: micro-focus sealed X-ray tube, PhotonJet (Cu) X-ray Source	3972 reflections with *I* > 2σ(*I*)
Mirror monochromator	*R*_int_ = 0.021
Detector resolution: 10.0000 pixels mm^-1^	θ_max_ = 78.0°, θ_min_ = 3.8°
ω scans	*h* = −12→16
Absorption correction: multi-scan (CrysAlisPro; Rigaku OD, 2021)	*k* = −15→15
*T*_min_ = 0.779, *T*_max_ = 1.000	*l* = −16→16
25388 measured reflections	

### Refinement

**Table:**

Refinement on *F*^2^	Primary atom site location: dual
Least-squares matrix: full	Hydrogen site location: mixed
*R*[*F*^2^ > 2σ(*F*^2^)] = 0.025	H atoms treated by a mixture of independent and constrained refinement
*wR*(*F*^2^) = 0.071	*w* = 1/[σ^2^(*F*_o_^2^) + (0.0427*P*)^2^ + 0.5759*P*] where *P* = (*F*_o_^2^ + 2*F*_c_^2^)/3
*S* = 1.09	(Δ/σ)_max_ = 0.001
4088 reflections	Δρ_max_ = 0.20 e Å^−^^3^
259 parameters	Δρ_min_ = −0.39 e Å^−^^3^
0 restraints	

### Special details

**Table:**

Geometry. All esds (except the esd in the dihedral angle between two l.s. planes) are estimated using the full covariance matrix. The cell esds are taken into account individually in the estimation of esds in distances, angles and torsion angles; correlations between esds in cell parameters are only used when they are defined by crystal symmetry. An approximate (isotropic) treatment of cell esds is used for estimating esds involving l.s. planes.

### Fractional atomic coordinates and isotropic or equivalent isotropic displacement parameters (Å^2^)

**Table:**

	*x*	*y*	*z*	*U*_iso_*/*U*_eq_	
Co1	0.500000	0.500000	0.500000	0.01162 (8)	
N1	0.49434 (8)	0.46285 (8)	0.65301 (8)	0.01532 (19)	
C1	0.46823 (9)	0.46250 (9)	0.72848 (10)	0.0135 (2)	
S1	0.43052 (3)	0.46382 (2)	0.83365 (3)	0.02096 (8)	
O1	0.62345 (7)	0.61232 (7)	0.58179 (7)	0.01480 (16)	
H1A	0.6441 (16)	0.6265 (15)	0.6543 (17)	0.034 (5)*	
H1B	0.6416 (17)	0.6677 (18)	0.5509 (18)	0.046 (6)*	
N11	0.36295 (8)	0.62692 (8)	0.47457 (8)	0.01335 (19)	
N12	0.31137 (9)	0.78613 (8)	0.55340 (9)	0.0178 (2)	
N13	0.23595 (9)	0.76282 (8)	0.34775 (9)	0.0188 (2)	
C11	0.32652 (10)	0.68571 (9)	0.36510 (10)	0.0175 (2)	
H11A	0.391355	0.722732	0.363717	0.021*	
H11B	0.300127	0.635132	0.303039	0.021*	
C12	0.40035 (10)	0.70880 (9)	0.56706 (10)	0.0163 (2)	
H12A	0.423172	0.673366	0.639612	0.020*	
H12B	0.465940	0.746048	0.567719	0.020*	
C13	0.27803 (11)	0.83883 (9)	0.44318 (11)	0.0194 (2)	
H13A	0.342809	0.876660	0.442661	0.023*	
H13B	0.219187	0.890515	0.432994	0.023*	
C14	0.21257 (10)	0.72912 (10)	0.55236 (11)	0.0194 (2)	
H14A	0.153212	0.779834	0.543355	0.023*	
H14B	0.233623	0.693517	0.624588	0.023*	
C15	0.13874 (10)	0.70598 (10)	0.35186 (10)	0.0196 (2)	
H15A	0.110306	0.654944	0.290289	0.023*	
H15B	0.078532	0.756406	0.340611	0.023*	
C16	0.25985 (9)	0.57494 (9)	0.47493 (10)	0.0156 (2)	
H16A	0.231942	0.522809	0.414419	0.019*	
H16B	0.280830	0.537541	0.546167	0.019*	
N14	0.16833 (8)	0.64994 (8)	0.46020 (9)	0.0173 (2)	
N21	0.70755 (8)	0.64919 (8)	0.80957 (8)	0.01507 (19)	
N22	0.71117 (9)	0.72608 (8)	0.98413 (9)	0.0167 (2)	
N23	0.87597 (9)	0.74634 (8)	0.93941 (9)	0.0184 (2)	
N24	0.83066 (8)	0.57193 (8)	0.99309 (8)	0.0165 (2)	
C21	0.64056 (10)	0.70037 (9)	0.86374 (10)	0.0166 (2)	
H21A	0.579657	0.653203	0.858804	0.020*	
H21B	0.606714	0.765088	0.823090	0.020*	
C22	0.75820 (10)	0.55052 (9)	0.87319 (10)	0.0159 (2)	
H22A	0.697996	0.502245	0.867830	0.019*	
H22B	0.802836	0.516073	0.838924	0.019*	
C23	0.76143 (11)	0.62520 (10)	1.04253 (10)	0.0184 (2)	
H23A	0.808248	0.639798	1.121947	0.022*	
H23B	0.700979	0.577825	1.038421	0.022*	
C24	0.80542 (11)	0.79507 (10)	0.98977 (11)	0.0195 (2)	
H24A	0.774373	0.861136	0.950611	0.023*	
H24B	0.852716	0.811921	1.068513	0.023*	
C25	0.92101 (10)	0.64522 (10)	0.99882 (10)	0.0197 (2)	
H25A	0.966605	0.611322	0.965300	0.024*	
H25B	0.969896	0.659937	1.077698	0.024*	
C26	0.80131 (10)	0.72159 (10)	0.82069 (10)	0.0180 (2)	
H26A	0.845870	0.689137	0.785169	0.022*	
H26B	0.769710	0.787150	0.780642	0.022*	
N31	0.02532 (10)	0.44658 (10)	0.24906 (11)	0.0297 (3)	
C31	−0.03061 (10)	0.46842 (10)	0.29362 (11)	0.0198 (2)	
C32	−0.10137 (13)	0.49834 (10)	0.35086 (13)	0.0242 (3)	
H32A	−0.179624	0.497188	0.297589	0.036*	
H32B	−0.081381	0.568548	0.381669	0.036*	
H32C	−0.089767	0.449026	0.411080	0.036*	

### Atomic displacement parameters (Å^2^)

**Table:**

	*U*^11^	*U*^22^	*U*^33^	*U*^12^	*U*^13^	*U*^23^
Co1	0.01283 (13)	0.01259 (14)	0.01083 (13)	−0.00162 (9)	0.00635 (10)	−0.00092 (9)
N1	0.0171 (5)	0.0161 (5)	0.0139 (5)	−0.0003 (4)	0.0077 (4)	0.0002 (4)
C1	0.0131 (5)	0.0120 (5)	0.0148 (5)	−0.0004 (4)	0.0052 (4)	−0.0002 (4)
S1	0.02595 (16)	0.02425 (17)	0.01980 (15)	0.00071 (11)	0.01660 (12)	−0.00011 (11)
O1	0.0176 (4)	0.0156 (4)	0.0124 (4)	−0.0034 (3)	0.0074 (3)	−0.0018 (3)
N11	0.0151 (4)	0.0126 (4)	0.0136 (4)	−0.0011 (3)	0.0072 (4)	−0.0004 (4)
N12	0.0189 (5)	0.0153 (5)	0.0207 (5)	0.0013 (4)	0.0099 (4)	−0.0024 (4)
N13	0.0229 (5)	0.0147 (5)	0.0199 (5)	0.0035 (4)	0.0099 (4)	0.0022 (4)
C11	0.0226 (6)	0.0159 (5)	0.0164 (5)	0.0025 (4)	0.0104 (5)	0.0022 (4)
C12	0.0164 (5)	0.0145 (5)	0.0178 (5)	0.0002 (4)	0.0071 (4)	−0.0040 (4)
C13	0.0232 (6)	0.0125 (5)	0.0245 (6)	0.0008 (4)	0.0120 (5)	0.0004 (4)
C14	0.0208 (6)	0.0197 (6)	0.0220 (6)	0.0026 (5)	0.0133 (5)	−0.0008 (5)
C15	0.0186 (5)	0.0175 (6)	0.0197 (6)	0.0023 (4)	0.0051 (5)	0.0012 (4)
C16	0.0140 (5)	0.0133 (5)	0.0201 (5)	0.0002 (4)	0.0078 (4)	0.0008 (4)
N14	0.0157 (5)	0.0156 (5)	0.0216 (5)	0.0024 (4)	0.0088 (4)	0.0013 (4)
N21	0.0186 (5)	0.0135 (4)	0.0142 (4)	0.0001 (4)	0.0080 (4)	−0.0003 (4)
N22	0.0219 (5)	0.0151 (5)	0.0170 (5)	−0.0011 (4)	0.0119 (4)	−0.0011 (4)
N23	0.0202 (5)	0.0178 (5)	0.0195 (5)	−0.0041 (4)	0.0107 (4)	−0.0024 (4)
N24	0.0174 (5)	0.0173 (5)	0.0153 (5)	−0.0001 (4)	0.0074 (4)	0.0009 (4)
C21	0.0172 (5)	0.0160 (5)	0.0178 (6)	0.0015 (4)	0.0084 (4)	0.0002 (4)
C22	0.0194 (5)	0.0129 (5)	0.0159 (5)	−0.0001 (4)	0.0079 (4)	−0.0011 (4)
C23	0.0240 (6)	0.0190 (6)	0.0159 (5)	0.0006 (5)	0.0120 (5)	0.0017 (4)
C24	0.0252 (6)	0.0159 (5)	0.0203 (6)	−0.0051 (5)	0.0124 (5)	−0.0059 (4)
C25	0.0162 (5)	0.0235 (6)	0.0189 (6)	−0.0017 (5)	0.0067 (4)	−0.0007 (5)
C26	0.0247 (6)	0.0171 (5)	0.0167 (6)	−0.0022 (4)	0.0130 (5)	0.0000 (4)
N31	0.0242 (6)	0.0341 (6)	0.0311 (6)	−0.0005 (5)	0.0119 (5)	−0.0095 (5)
C31	0.0180 (6)	0.0164 (6)	0.0216 (6)	0.0005 (5)	0.0051 (5)	−0.0009 (5)
C32	0.0226 (7)	0.0262 (7)	0.0267 (7)	0.0014 (5)	0.0131 (6)	0.0011 (5)

### Geometric parameters (Å, º)

**Table:**

Co1—N1^i^	2.0744 (10)	C16—H16B	0.9700
Co1—N1	2.0744 (10)	C16—N14	1.4670 (14)
Co1—O1^i^	2.0661 (8)	N21—C21	1.4786 (14)
Co1—O1	2.0661 (8)	N21—C22	1.4840 (14)
Co1—N11	2.3112 (9)	N21—C26	1.4797 (15)
Co1—N11^i^	2.3112 (9)	N22—C21	1.4788 (15)
N1—C1	1.1654 (16)	N22—C23	1.4827 (15)
C1—S1	1.6352 (12)	N22—C24	1.4784 (15)
O1—H1A	0.88 (2)	N23—C24	1.4669 (15)
O1—H1B	0.88 (2)	N23—C25	1.4763 (16)
N11—C11	1.4931 (14)	N23—C26	1.4695 (15)
N11—C12	1.5008 (14)	N24—C22	1.4673 (15)
N11—C16	1.4934 (14)	N24—C23	1.4697 (15)
N12—C12	1.4645 (14)	N24—C25	1.4707 (15)
N12—C13	1.4690 (16)	C21—H21A	0.9700
N12—C14	1.4665 (15)	C21—H21B	0.9700
N13—C11	1.4685 (15)	C22—H22A	0.9700
N13—C13	1.4773 (16)	C22—H22B	0.9700
N13—C15	1.4727 (16)	C23—H23A	0.9700
C11—H11A	0.9700	C23—H23B	0.9700
C11—H11B	0.9700	C24—H24A	0.9700
C12—H12A	0.9700	C24—H24B	0.9700
C12—H12B	0.9700	C25—H25A	0.9700
C13—H13A	0.9700	C25—H25B	0.9700
C13—H13B	0.9700	C26—H26A	0.9700
C14—H14A	0.9700	C26—H26B	0.9700
C14—H14B	0.9700	N31—C31	1.1377 (18)
C14—N14	1.4764 (16)	C31—C32	1.4554 (18)
C15—H15A	0.9700	C32—H32A	0.9600
C15—H15B	0.9700	C32—H32B	0.9600
C15—N14	1.4740 (15)	C32—H32C	0.9600
C16—H16A	0.9700		
			
N1^i^—Co1—N1	180.0	N11—C16—H16B	108.9
N1—Co1—N11^i^	92.49 (4)	H16A—C16—H16B	107.7
N1^i^—Co1—N11^i^	87.51 (4)	N14—C16—N11	113.40 (9)
N1—Co1—N11	87.51 (4)	N14—C16—H16A	108.9
N1^i^—Co1—N11	92.49 (4)	N14—C16—H16B	108.9
O1—Co1—N1^i^	90.32 (4)	C15—N14—C14	107.93 (9)
O1^i^—Co1—N1^i^	89.68 (4)	C16—N14—C14	108.13 (9)
O1—Co1—N1	89.68 (4)	C16—N14—C15	107.69 (9)
O1^i^—Co1—N1	90.32 (4)	C21—N21—C22	108.15 (9)
O1^i^—Co1—O1	180.0	C21—N21—C26	108.13 (9)
O1—Co1—N11	89.16 (3)	C26—N21—C22	107.90 (9)
O1^i^—Co1—N11	90.84 (3)	C21—N22—C23	107.32 (9)
O1—Co1—N11^i^	90.84 (3)	C24—N22—C21	108.26 (9)
O1^i^—Co1—N11^i^	89.16 (3)	C24—N22—C23	107.49 (9)
N11^i^—Co1—N11	180.0	C24—N23—C25	108.18 (9)
C1—N1—Co1	161.53 (9)	C24—N23—C26	107.26 (9)
N1—C1—S1	179.08 (11)	C26—N23—C25	107.92 (9)
Co1—O1—H1A	120.6 (12)	C22—N24—C23	108.11 (9)
Co1—O1—H1B	127.1 (14)	C22—N24—C25	108.12 (9)
H1A—O1—H1B	107.8 (18)	C23—N24—C25	108.33 (9)
C11—N11—Co1	113.28 (7)	N21—C21—N22	111.82 (9)
C11—N11—C12	106.78 (9)	N21—C21—H21A	109.3
C11—N11—C16	107.17 (9)	N21—C21—H21B	109.3
C12—N11—Co1	112.96 (7)	N22—C21—H21A	109.3
C16—N11—Co1	109.60 (7)	N22—C21—H21B	109.3
C16—N11—C12	106.68 (8)	H21A—C21—H21B	107.9
C12—N12—C13	108.19 (9)	N21—C22—H22A	109.2
C12—N12—C14	108.65 (9)	N21—C22—H22B	109.2
C14—N12—C13	108.33 (10)	N24—C22—N21	111.96 (9)
C11—N13—C13	108.05 (9)	N24—C22—H22A	109.2
C11—N13—C15	108.54 (9)	N24—C22—H22B	109.2
C15—N13—C13	107.73 (9)	H22A—C22—H22B	107.9
N11—C11—H11A	109.0	N22—C23—H23A	109.1
N11—C11—H11B	109.0	N22—C23—H23B	109.1
N13—C11—N11	112.72 (9)	N24—C23—N22	112.71 (9)
N13—C11—H11A	109.0	N24—C23—H23A	109.1
N13—C11—H11B	109.0	N24—C23—H23B	109.1
H11A—C11—H11B	107.8	H23A—C23—H23B	107.8
N11—C12—H12A	109.0	N22—C24—H24A	108.9
N11—C12—H12B	109.0	N22—C24—H24B	108.9
N12—C12—N11	112.74 (9)	N23—C24—N22	113.15 (9)
N12—C12—H12A	109.0	N23—C24—H24A	108.9
N12—C12—H12B	109.0	N23—C24—H24B	108.9
H12A—C12—H12B	107.8	H24A—C24—H24B	107.8
N12—C13—N13	112.28 (10)	N23—C25—H25A	109.1
N12—C13—H13A	109.1	N23—C25—H25B	109.1
N12—C13—H13B	109.1	N24—C25—N23	112.47 (10)
N13—C13—H13A	109.1	N24—C25—H25A	109.1
N13—C13—H13B	109.1	N24—C25—H25B	109.1
H13A—C13—H13B	107.9	H25A—C25—H25B	107.8
N12—C14—H14A	109.2	N21—C26—H26A	109.1
N12—C14—H14B	109.2	N21—C26—H26B	109.1
N12—C14—N14	112.24 (9)	N23—C26—N21	112.70 (9)
H14A—C14—H14B	107.9	N23—C26—H26A	109.1
N14—C14—H14A	109.2	N23—C26—H26B	109.1
N14—C14—H14B	109.2	H26A—C26—H26B	107.8
N13—C15—H15A	109.1	N31—C31—C32	178.95 (14)
N13—C15—H15B	109.1	C31—C32—H32A	109.5
N13—C15—N14	112.61 (10)	C31—C32—H32B	109.5
H15A—C15—H15B	107.8	C31—C32—H32C	109.5
N14—C15—H15A	109.1	H32A—C32—H32B	109.5
N14—C15—H15B	109.1	H32A—C32—H32C	109.5
N11—C16—H16A	108.9	H32B—C32—H32C	109.5
			
Co1—N11—C11—N13	177.50 (7)	C16—N11—C12—N12	−56.82 (12)
Co1—N11—C12—N12	−177.30 (7)	C21—N21—C22—N24	−58.62 (12)
Co1—N11—C16—N14	179.51 (7)	C21—N21—C26—N23	59.00 (12)
N11—C16—N14—C14	−58.13 (12)	C21—N22—C23—N24	58.82 (12)
N11—C16—N14—C15	58.26 (12)	C21—N22—C24—N23	−58.16 (13)
N12—C14—N14—C15	−58.00 (12)	C22—N21—C21—N22	58.99 (12)
N12—C14—N14—C16	58.24 (12)	C22—N21—C26—N23	−57.76 (12)
N13—C15—N14—C14	58.10 (12)	C22—N24—C23—N22	−58.82 (12)
N13—C15—N14—C16	−58.43 (12)	C22—N24—C25—N23	59.03 (12)
C11—N11—C12—N12	57.52 (12)	C23—N22—C21—N21	−58.65 (12)
C11—N11—C16—N14	−57.18 (12)	C23—N22—C24—N23	57.48 (12)
C11—N13—C13—N12	−58.92 (12)	C23—N24—C22—N21	58.22 (12)
C11—N13—C15—N14	58.66 (12)	C23—N24—C25—N23	−57.90 (12)
C12—N11—C11—N13	−57.52 (12)	C24—N22—C21—N21	57.10 (12)
C12—N11—C16—N14	56.90 (12)	C24—N22—C23—N24	−57.44 (12)
C12—N12—C13—N13	58.99 (12)	C24—N23—C25—N24	57.61 (12)
C12—N12—C14—N14	−58.86 (12)	C24—N23—C26—N21	−58.72 (12)
C13—N12—C12—N11	−58.73 (12)	C25—N23—C24—N22	−57.80 (13)
C13—N12—C14—N14	58.46 (12)	C25—N23—C26—N21	57.63 (12)
C13—N13—C11—N11	58.71 (12)	C25—N24—C22—N21	−58.86 (12)
C13—N13—C15—N14	−58.10 (12)	C25—N24—C23—N22	58.11 (12)
C14—N12—C12—N11	58.67 (12)	C26—N21—C21—N22	−57.60 (12)
C14—N12—C13—N13	−58.62 (12)	C26—N21—C22—N24	58.13 (11)
C15—N13—C11—N11	−57.84 (12)	C26—N23—C24—N22	58.38 (13)
C15—N13—C13—N12	58.16 (12)	C26—N23—C25—N24	−58.14 (12)
C16—N11—C11—N13	56.49 (12)		

Symmetry code: (i) −*x*+1, −*y*+1, −*z*+1.

### Hydrogen-bond geometry (Å, º)

**Table:**

*D*—H···*A*	*D*—H	H···*A*	*D*···*A*	*D*—H···*A*
O1—H1*A*···N21	0.88 (2)	1.85 (2)	2.7298 (13)	171.8 (18)
O1—H1*B*···N22^ii^	0.88 (2)	2.01 (2)	2.8759 (13)	167.7 (19)
C12—H12*B*···O1	0.97	2.60	3.0752 (14)	111
C13—H13*A*···S1^ii^	0.97	2.95	3.8089 (12)	148
C13—H13*B*···N24^iii^	0.97	2.66	3.5045 (16)	146
C16—H16*A*···O1^i^	0.97	2.52	3.0571 (14)	115
C16—H16*B*···N1	0.97	2.70	3.2713 (15)	118
C21—H21*A*···S1	0.97	3.00	3.9471 (12)	165
C23—H23*B*···S1^iv^	0.97	2.89	3.6683 (12)	138
C26—H26*A*···N31^i^	0.97	2.56	3.4794 (17)	158
C32—H32*A*···S1^v^	0.96	3.02	3.9560 (15)	166
C32—H32*B*···N23^vi^	0.96	2.58	3.4685 (16)	154
C32—H32*C*···N14^v^	0.96	2.61	3.4750 (17)	149

Symmetry codes: (i) −*x*+1, −*y*+1, −*z*+1; (ii) *x*, −*y*+3/2, *z*−1/2; (iii) −*x*+1, *y*+1/2, −*z*+3/2; (iv) −*x*+1, −*y*+1, −*z*+2; (v) −*x*, −*y*+1, −*z*+1; (vi) *x*−1, −*y*+3/2, *z*−1/2.
