# Supplementary figures and images for: Synthesis and crystal structure of di­aqua­bis­(hexa­methyl­enetramine-κN)bis­(thio­cyanato-κN)cobalt(II)–hexa­methyl­ene­tetra­mine–aceto­nitrile (1/2/2)
Source: Acta Crystallogr E Crystallogr Commun. 2021 Oct 8;77(Pt 11):1082–6. doi: 10.1107/S2056989021010033 (PMC8587985; doi:10.1107/S2056989021010033)

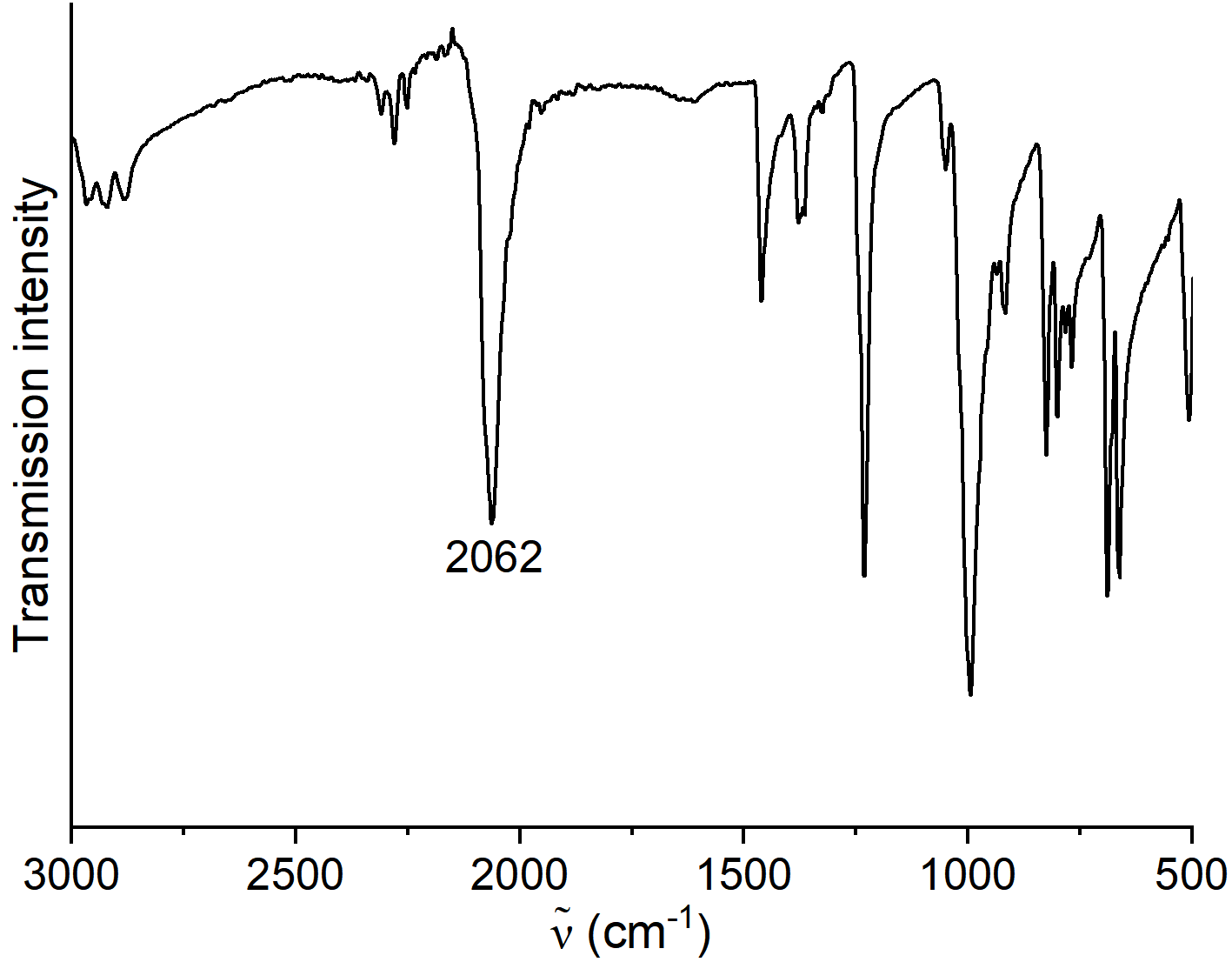

Supplement: Supplementary file 3 [file e-77-01082-sup3.png]

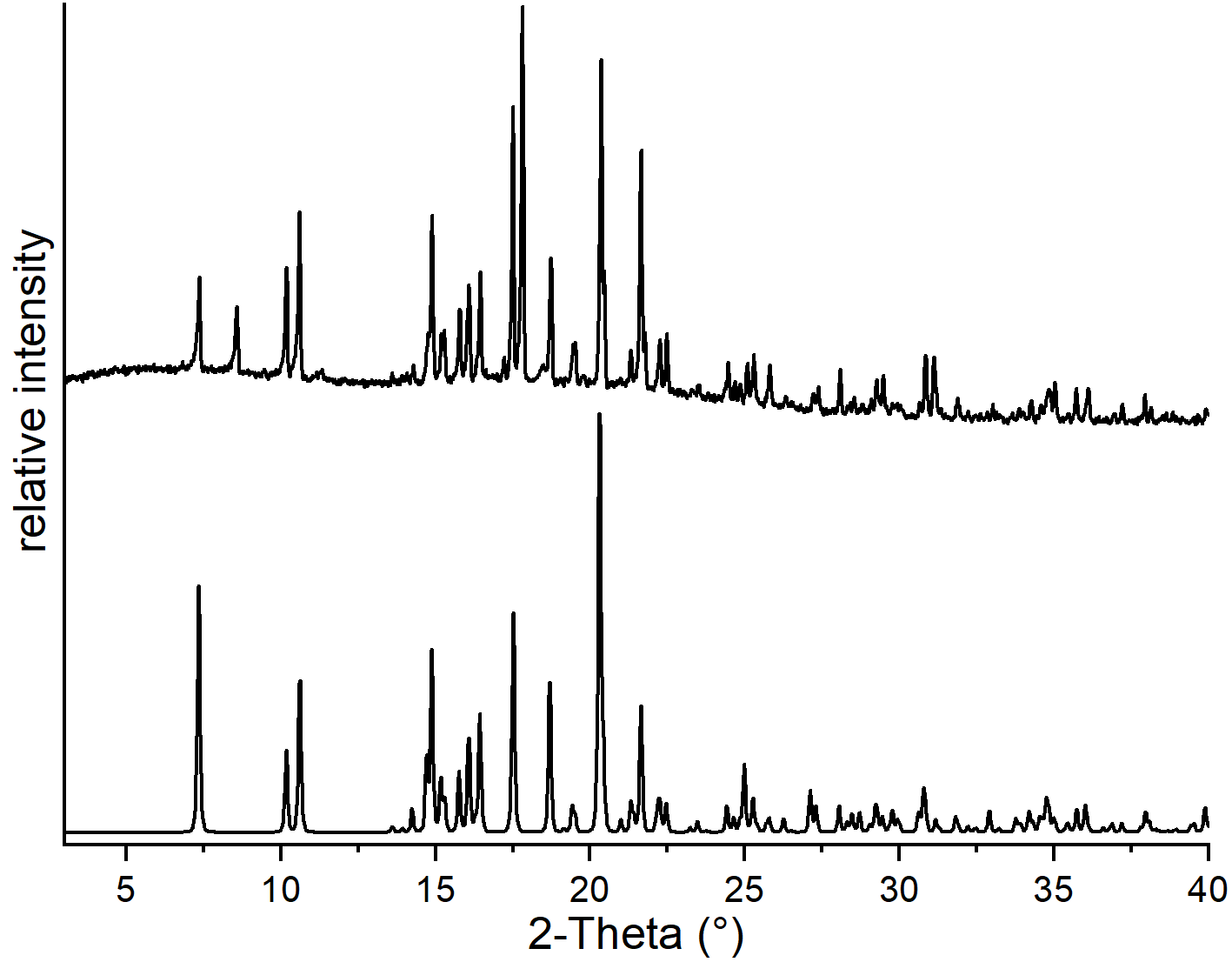

Supplement: Supplementary file 4 [file e-77-01082-sup4.png]
